# Robotic natural orifice specimen extraction surgery versus conventional robotic resection for patients with colorectal neoplasms

**DOI:** 10.3389/fonc.2023.1153751

**Published:** 2023-03-17

**Authors:** Linye Li, Kuijie Liu, Tiegang Li, Jiangjiao Zhou, Shu Xu, Nanhui Yu, Zhushu Guo, Hongliang Yao

**Affiliations:** ^1^ Department of General Surgery, The Second Xiangya Hospital of Central South University, Changsha, China; ^2^ Department of Gastrointestinal Surgery, The Second Xiangya Hospital of Central South University, Changsha, China; ^3^ Department of Biliary and Pancreatic Surgery, The Second Xiangya Hospital of Central South University, Changsha, China

**Keywords:** colorectal neoplasms, robotic surgery, natural orifice specimen extraction, surgical outcomes, survival outcomes

## Abstract

**Background:**

Laparoscopic natural orifice specimen extraction surgery (NOSES) has been widely used in colorectal neoplasms. However, only a few studies have focused on robotic NOSES. This study compared the short-term clinical outcomes and long-term survival outcomes between robotic NOSES and conventional robotic resection (CRR) groups.

**Methods:**

From March 2016 to October 2018, a consecutive of 143 patients who underwent robotic sigmoid and rectal resection at the Department of Gastrointestinal Surgery, The Second Xiangya Hospital, Central South University, were considered for inclusion in this study. Propensity-score matching (PSM) was conducted to account for differences in the baseline characteristics. After PSM, 39 patients were included in the robotic NOSES group, and 39 patients in the CRR group. The baseline characteristics between the two groups were all balanced and comparable.

**Results:**

Patients in the NOSES group experienced less intraoperative blood loss (p=0.001), lower requirements for additional analgesia (p=0.020), shorter time to first flatus (p=0.010), and a shorter time to first liquid diet (p=0.003) than the CRR group. The 3-year overall survival rates (NOSES: 92.3% vs. CRR: 89.7% p=1.000) and 3-year disease-free survival rates (NOSES: 82.1% vs. CRR: 84.6% p=0.761) between the two groups were comparable.

**Conclusion:**

Robotic natural orifice specimen extraction surgery is a safe and feasible surgery for patients with colorectal neoplasms. Robotic NOSES is associated with better short-term clinical outcomes and similar long-term survival outcomes to conventional robotic resection.

## Introduction

Minimally invasive surgery, including laparoscopic and robotic surgery, has been widely used in patients with colorectal cancer (1). However, the incision made at the abdominal wall to take out the specimen could develop complications such as wound infection, postoperative pain, and incisional hernia (2). Therefore, to prevent these complications, natural orifice specimen extraction (NOSE) surgery, in which the specimen is removed through a natural orifice (vagina or anus), has been considered a safe and feasible alternative in patients with a tumor diameter ≤5.0cm and body mass index<30 kg/m² (3). Robotic surgery is advantageous over laparoscopic surgery as it offers better visualization, a stable camera platform, stabilization of tremors, and greater dexterity of movements (4). Although the safety and feasibility of laparoscopic NOSES in colorectal surgery have been well proven (5–7), only a few studies have compared the long-term outcomes of robotic natural orifice specimen extraction surgery and conventional robotic resection in the treatment of colorectal tumors. Since our center performs robotic NOSES (8), we conducted a single-center, retrospective study to investigate the safety and feasibility of robotic NOSES.

## Patients and methods

### Patients

**Figure 1:**
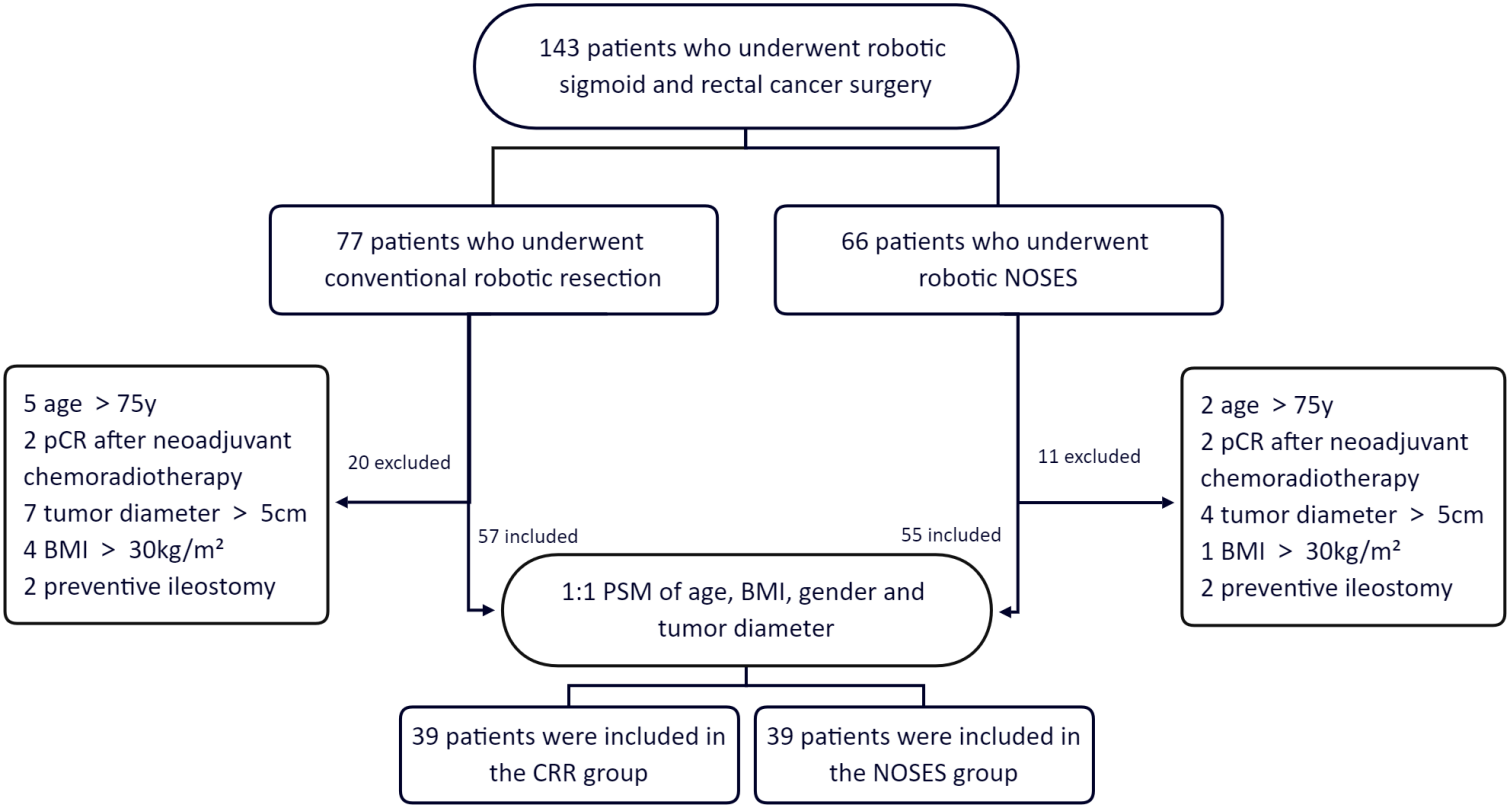
Group information. pCR pathological complete response. BMI body mass index. PSM propensity-score matching.

From March 2016 to October 2018, a consecutive of 143 patients underwent robotic sigmoid and rectal colon cancer surgery at the Department of Gastrointestinal Surgery, The Second Xiangya Hospital, Central South University. Patients who underwent robotic natural orifice specimen extraction surgery were assigned to the NOSES group, and patients who underwent conventional robotic resection were assigned to the CRR group. NOSES can either be transanal or transvaginal. However, our center only performed transanal NOSES. The inclusion criteria of this study were (1): Age:18-75 years old; (2) body mass index (BMI) <30 kg/m^2^; (3) no distant metastases; (4) T2, T3 and T4 rectal cancers; and (5) tumor diameter ≤5cm. The exclusion criteria were: (1) contraindications for robotic surgery; (2) patients with intestinal obstruction or perforation; (3) patients who underwent preventive ileostomy; (4) benign disease, and (5) pathological complete response (pCR) after neoadjuvant chemoradiotherapy. Based on the eligibility criteria, 55 of the 66 patients who underwent robotic NOSES and 57 of the 77 patients who underwent conventional robotic resection were included in this study. Propensity-score matching (PSM 1:1) was performed to account for differences in age, BMI, gender, and tumor diameter. The propensity-score matching yielded 39 patients in the NOSES group and 39 in the CRR group (**Figure 1**).

### Interventions

The guidelines of the National Comprehensive Cancer Network (NCCN) were used for perioperative management. Colorectal cancer was diagnosed by physical examinations, colonoscopic biopsies and radiographic examinations, including abdominal computed tomography scan and pelvic magnetic resonance imaging. Further, T and N staging of colorectal cancer was done using the American Joint Committee on Cancer (AJCC, eighth edition). Patients with cT3+, N+, or low-lying rectal cancer who desired to preserve the anus were advised by the multi-disciplinary team to receive preoperative chemoradiotherapy. Limited by the local economic level local socio-economic factors, few patients received preoperative neoadjuvant chemoradiotherapy. All operations were performed by Prof. Yao and Prof. Li, who are highly experienced in robotic colorectal cancer surgeries. Patient-controlled analgesia (PCA) was used to manage postoperative pain.

### Ethics

This study was conducted in compliance with the Declaration of Helsinki, and the protocol was approved by the Ethics Committee of the Second Xiangya Hospital, Central South University. Further, all patients signed written informed consents before being operated.

### Surgical procedure

**Figure 2:**
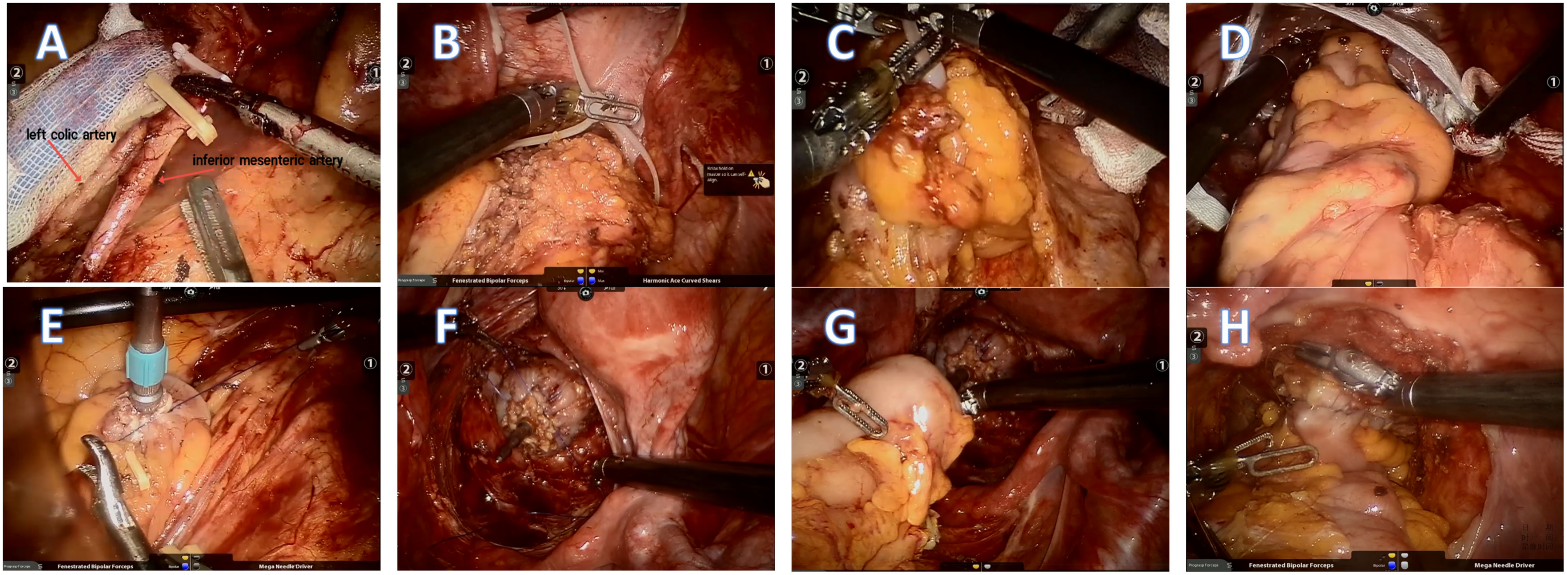
**(A )**The inferior mesenteric artery and vein were isolated and clipped by absorbable vascular clamps. However, the left colic artery was preserved. **(B, C)** The proximal and distal colorectum were ligated with a self-locking nylon bandage at 2-5cm to the edge of the tumor and then cut off by an ultrasonic scalpel. **(D)** The resected specimen and endoscope-sterile sleeve were pulled out through the anus. **(E, F)** Double purse-string sutures were made. **(G, H)** The anastomosis was completed and strengthened by running suture.

After the successful induction of anesthesia, the patient was placed in a Trendelenburg position. Five trocars (three 8mm and two 12mm) were used for the surgery. Trocar R1 (ultrasonic knife, 8mm) was placed at the right McBurney’s point. Trocar R2 (bipolar electrocoagulation, 8mm) was placed at the left midclavicular line 1 cm above the umbilicus. Further, trocar R3 (auxiliary grasping forceps, 8mm) was placed at the left anterior superior iliac spine. A 12mm trocar placed 3 cm above the umbilicus was used for the robotic camera, while the other 12mm trocar placed at the right midclavicular line 1 cm above the umbilicus was used for the assistant’s forceps placement. First, the surgeons explored the abdominal cavity to determine whether the tumor had invaded the adjacent tissues and organs or undergone distant metastasis. The inferior mesenteric artery and vein were isolated and clipped by absorbable vascular clamps while protecting the reproductive tract vessels and ureters. However, the left colic artery was preserved. The surgeons ensured that the colon length was enough to enable tension-free anastomosis. The proximal and distal colorectum were ligated with a self-locking nylon bandage at 2-5cm to the edge of the tumor and then cut off by an ultrasonic scalpel. If the tumor site was low,the surgeons performed single anastomosis using a circular stapler (9). The assistant sent an anvil through the anus, and the operator made a purse-string suture of the stump of the sigmoid colon and placed the anvil into the sigmoid colon. After that, the assistant placed the curved intraluminal stapler through the anus to complete the anastomosis. If the tumor site was comparatively higher, a double-anastomosis was made and the anastomosis was strengthened by an embedded suture at the bilateral stapled corners made by the double-anastomos if possible. For the NOSES group, an assistant inserted an endoscope-sterile sleeve for the protection of the specimen through the rectum enteric cavity, which had been disinfected with povidone-iodine and sterile saline. The resected specimen was pulled out through the anus. For the CRR group, a 5cm incision was made in the hypogastrium, through which the specimen was pulled out. For both groups, after confirming that the anastomosis was satisfactory by the air leak test, the mesenteric hole and pelvic floor peritoneum were closed. Finally, the abdomen was closed after indwelling an anterior sacral pelvic drainage tube (**Figure 2**).

### Follow up

Patients with T3 and T4 colorectal cancer or N+ received postoperative chemotherapy. The patients were followed up through the outpatient department every three months for three years after the surgery. Physical examinations and laboratory investigations, including digital rectal exam, CEA, and CA 19-9, were carried out at every follow-up. CT enhancement scans of the chest, abdomen, and pelvis were performed every six months. In addition, a colonoscopy was done at least once a year. Patients living far from our hospital were followed up at the nearest hospital and had their results sent electronically. Tumor recurrence and metastasis were managed by a multidisciplinary team in collaboration. All patients were followed until death or till the end of the study (July 2022). The long-term endpoints were 3-year overall survival (OS) and disease-free survival (DFS).

### Statistical analysis

Data were analyzed using SPSS software (version 26). Propensity-score matching (PSM) was used to adjust for differences in the baseline data, including age, gender, BMI, and tumor diameter. Quantitative data were expressed as mean ± standard deviation and compared by the independent sample t-test. However, qualitative data were compared using the χ^2^ or Fisher’s exact test. The Kaplan–Meier method was used to compare survival between the two groups. Differences in survival were compared using the log-rank test. A P-value <0.05 was considered statistically significant.

## Results

### Clinical characteristics

A total of 78 patients were included in this study after the propensity-score matching. The results revealed significant differences in T stage and tumor diameter before the propensity-score matching between the NOSES (p=0.041) and CRR (p=0.032) groups. However, after propensity-score matching, there were no significant differences between the two groups (**Table 1**).

**Table 1:** Clinical characteristics. p<0.05 are indicated in red.

Characteristics	Before PSM			After PSM		
	Conventional Robotic resection (N=57)	Robotic NOSES (N=55)	P.value	Conventional Robotic resection (N=39)	Robotic NOSES (N=39)	P.value
Gender						
Male	35 (61.4%)	33 (60.0%)	0.879	25 (64.1%)	23 (59.0%)	0.642
female	22 (38.6%)	22 (40.0%)		14 (35.9%)	16 (41.0%)	
Age (years)	56.0 ± 10.4	57.7 ± 10.3	0.384	55.2 ± 11.3	57.7 ± 10.3	0.304
BMI (Kg/m^2^)	22.8 ± 2.8	22.5 ± 2.7	0.656	22.6 ± 3.1	22.5 ± 2.5	0.855
ASA score						
I	4 (7.0%)	3 (5.5%)	0.908	3 (7.7%)	1 (2.6%)	0.500
II	35 (61.4%)	36 (65.5%)		24 (61.5%)	28 (71.8%)	
III	18 (31.6%)	16 (29.0%)		12 (30.8%)	10 (25.6%)	
CDmax(cm)	3.9 ± 1.1	3.5 ± 1.1	0.032	3.7 ± 1.1	3.8 ± 1.0	0.775
Distence from tumor to anus(cm)	13.3 ± 5.9	12.3 ± 5.6	0.340	13.1 ± 5.5	13.1 ± 5.7	1.000
Abdominal surgery history						
+	7(12.3%)	12 (21.8%)	0.179	3 (7.7%)	9 (23.1%)	0.060
–	50(87.7%)	43 (78.2%)		36 (92.3%)	30 (76.9)	
Preoperative chemoradiotherapy						
+	3 (3.4%)	3 (5.8%)	1.000	3 (7.7%)	2 (5.1%)	1.000
–	54 (96.6%)	52 (94.2%)		36 (92.3%)	37 (94.9%)	
Preoperative serum CEA(ng/ml)	7.1 ± 11.5	4.5 ± 8.7	0.182	6.7 ± 13.2	3.3 ± 4.1	0.129
Preoperative serum CA19-9(KU/L)	15.4 ± 26.3	13.3 ± 16.4	0.606	14.9 ± 23.8	14.4 ± 19.2	0.909
T stage						
T2	11(19.3%)	21(38.1%)	0.041	11(28.2%)	14(35.9%)	0.433
T3	22(38.6%)	21(38.1%)		15(38.5%)	17(43.6%)	
T4	24(42.1%)	13(23.8%)		13(34.3%)	8(20.5%)	
N0	31 (54.4%)	37 (67.3%)	0.236	21 (53.9%)	27 (69.2%)	0.279
N1	15 (26.3%)	13 (23.6%)		11 (28.2%)	9 (23.1%)	
N2	11 (19.3%)	5 (9.1%)		7 (17.9%)	3 (7.7%)	
Histological differentiation						
Well	7 (12.3%)	7 (12.7%)	0.498	7 (17.9%)	2 (5.1%)	0.163
Moderate	38 (66.7%)	41 (74.6%)		26 (66.7%)	33 (84.6%)	
Poor	12 (21.0%)	7 (12.7)		6 (15.4%)	4 (13.3%)	

BMI body mass index, ASA score American society of anesthesiologists score.

“+” means “positive” and “-” means “negative”.

### Short-term clinical outcomes

All surgeries were successfully performed with no conversion to open surgery. There were no significant differences in operation time between the two groups (NOSES 181.3 ± 43.8 min vs. CRR 193.1 ± 46.9 min, p=0.256). Furthermore, there were no significant differences in the number of harvested lymph nodes between the two groups (NOSES 16.3 ± 4.5 vs. CRR 15.9 ± 4.6 p=0.691). However, the NOSES group had significantly less intraoperative blood loss than the CRR group (41.3 ± 18.7 ml vs. 67.3 ± 44.3 ml p=0.001). Moreover, the NOSES group showed superior postoperative gastrointestinal function recovery compared with the CRR group, The time to first flatus between the NOSES and CRR groups was 3.1 ± 0.8 d vs. 3.6 ± 1.0 d (p=0.010), while the time to first liquid diet was (4.1 ± 0.8 d vs. 5.1 ± 1.8 d (p=0.003). Moreover, patients in the NOSES group suffered less severe pain, with lower requirements for additional analgesia than patients in the CRR group (15.4% vs. 38.5% p=0.002). Postoperative complications occurred in three patients in the NOSES group and five in the CRR group (p=0.709). There were no reported wound infections in the NOSES group, while there were two reported cases of wound infections in the CRR group. One patient in the NOSES group suffered from an intra-abdominal abscess that was managed by peritoneal drainage. There were two cases of anastomotic leakage (one in the NOSES group and one in the CRR group), which were managed by negative pressure suction and administration of antibiotics. One patient in the CRR group underwent reoperation due to intraperitoneal bleeding. One patient in the NOSES group and two in the CRR group developed pneumonia. There were no significant differences in the white blood cell count on postoperative days one and three between the two groups (p=0.601 and p=0.243, respectively) (**Table 2**).

**Table 2:** Short-term clinical outcomes. p<0.05 are indicated in red.

Outcome	After PSM		
	Conventional Robotic resection (N=39)	Robotic NOSES (N=39)	P.value
Operation time (mins)	193.1 ± 46.9	181.3 ± 43.8	0.256
Estimated blood loss(ml)	67.3 ± 44.3	41.3 ± 18.7	0.001
Additional analgesia require			
+	15 (38.5%)	6 (15.4%)	0.020
–	24 (61.5%)	33 (84.5%)	
Time to first flatus (d)	3.6 ± 1.0	3.1 ± 0.8	0.010
Time to first liquid diet (d)	5.1 ± 1.8	4.1 ± 0.8	0.003
Postoperative hospital stay (d)	9.5 ± 3.3	8.8 ± 2.9	0.364
Postoperative complications			
–	34	36	0.709
+	5	3	
Anastomotic leakage	1	1	
Bleeding	1	0	
Wound infection	2	0	
Intra-abdominal abscess	0	1	
Ileus	0	0	
Pneumonia	2	1	
Harvested lymph nodes	15.9 ± 4.6	16.3 ± 4.5	0.691
WBC count difference (/L)			
POD1	11.4 ± 3.7	11.0 ± 3.1	0.601
POD3	8.0 ± 2.7	7.4 ± 2.0	0.243

WBC white blood cell. POD1 post operation day one. POD3 post operation day three.

“+” means “positive” and “-” means “negative”.

### Long-term survival outcomes

**Figure 3:**
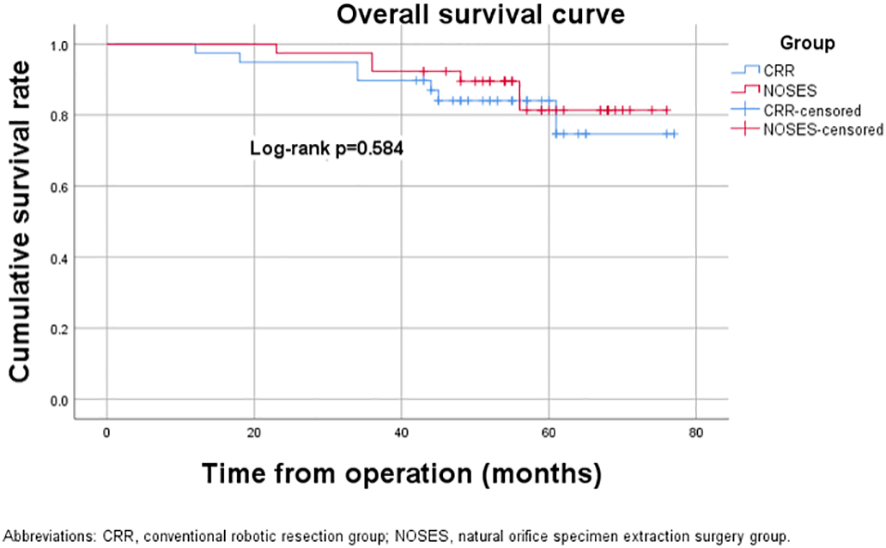
Overall survival curve. CRR, conventional robotic resection group. NOSES, natural orifice specimen extraction surgery group.

**Figure 4:**
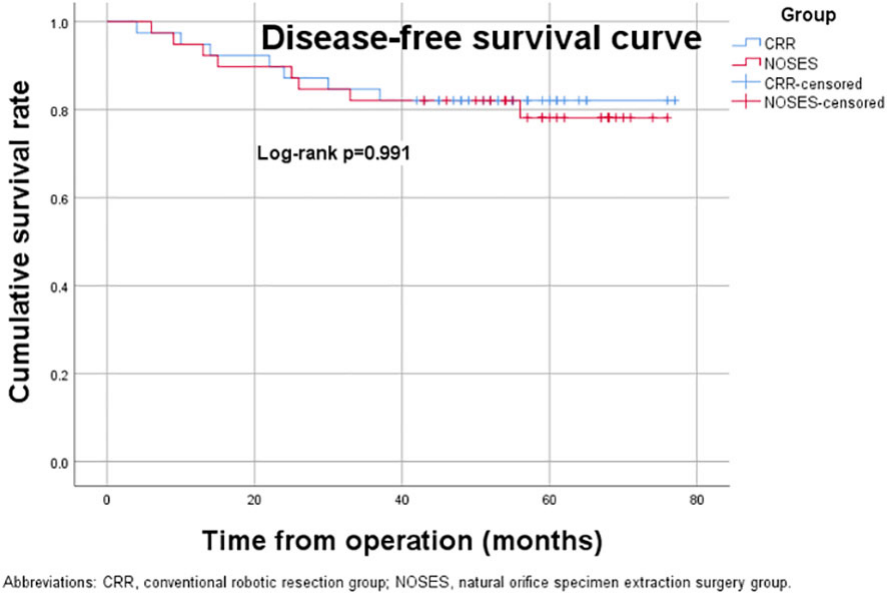
Disease-free survival curve. CRR, conventional robotic resection group. NOSES, natural orifice specimen extraction surgery group.

The median follow-up period was 59.0 (range, 23-76) months in the NOSES group and 55.0 (range, 12-77) months in the CRR group. During the follow-up period, 13 of the 78 patients died, while 14 had a local recurrence or distant metastasis (**Table 3**). In the NOSES group, seven patients developed recurrence or metastasis, and five died at 23, 36, 36, 48, and 56 months. However, six of the seven patients who developed recurrence or metastasis in the CRR group died at 12, 18, 23, 34, 44, and 45 months. Moreover, one patient in the NOSES group and one in the CRR group died due to other reasons. The Kaplan–Meier survival curves and the log-rank tests showed no significant difference in OS (p=0.584) and DFS (p=0.991) between the two groups (**Figures 3**, **4**). Furthermore, there were no significant differences in the 3-years OS and DFS between the two groups (OS: NOSES 92.3% vs. CRR 89.7%, p=1.000; DFS: NOSES 82.1% vs. CRR 84.6%, p=0.761)

**Table 3:** Recurrence and end points.

Case	Group	Gender	Stage	Time of recurrence (months)	Site of recurrence	Time of death (months)
1	NOSES	M	T3N0	25	Liver	/
2	NOSES	M	T3N1	15	Liver	23
3	NOSES	F	T4N0	33	Liver	56
4	NOSES	F	T4N0	26	Liver	48
5	NOSES	F	T4N0	6	Abdominal pelvic cavity	36
6	NOSES	M	T3N2	13	Local lymph node	36
7	NOSES	F	T3N1	/	/	56 (Died due to other reasons)
8	NOSES	M	T2N1	9	Lung	/
9	CRR	F	T2N1	22	Vagina	44
10	CRR	M	T3N0	30	Lung	34
11	CRR	M	T4N2	10	Liver	23
12	CRR	F	T4N2	24	Uterine accessories	45
13	CRR	F	T4N2	14	Abdominal pelvic cavity	18
14	CRR	M	T4N0	37	Liver	/
15	CRR	M	T3N2	4	Liver	12
16	CRR	M	T3N0	/	/	53 (Died due to other reasons)

“/” means the patient did not have a recurrence or did not die till the end of the study (July 2022). F, Female; M, Male.

## Discussion

Over the last 20 years, laparoscopic colorectal surgery has shown better postoperative outcomes, including lower incidences of postoperative morbidity and shorter hospital stays than open surgery. Further, laparoscopic natural orifice specimen extraction surgery (NOSES) was shown to have lower complications, including reduced postoperative pain and wound infections, than conventional laparoscopic surgery. However, the 2019 International consensus on NOSES for colorectal cancer has raised several potential pitfalls, such as bacteriological concerns, oncological outcomes, and patient selection (10). Several studies have demonstrated the safety and feasibility of laparoscopic NOSES (11–13). According to Ouyang et al. (14), unlike conventional laparoscopic surgery, NOSES did not increase postoperative pelvic and abdominal infections or promote tumor cell planting and metastasis. Most previous studies focused on laparoscopic NOSES, with a few focusing on robotic NOSES. In this study, we investigated the safety and feasibility of robotic NOSES in colorectal cancer since our center is one of the centers that conduct robotic NOSES in China (8, 9). Most previous studies on robotic NOSES did not explore long-term survival outcomes. Therefore, this study focused on the short-term clinical outcomes and long-term survival outcomes of robotic NOSES compared to conventional robotic resection. We employed propensity-score matching to account for differences in the baseline data.

Patients who underwent robotic NOSES experienced less blood loss ((41.3 ± 18.7 ml vs. 67.3 ± 44.3 ml p=0.001), less pain, and faster gastrointestinal function recovery than those who underwent conventional robotic resection (CRR). However, the two groups had no significant differences in postoperative complications. The robotic NOSES group did not report any wound infections or bleeding cases. According to Tang et al. (11), less blood loss in the NOSES group was because NOSES do not require auxiliary incisions. Furthermore, the NOSES procedure was performed more meticulously and gently under full laparoscopic surveillance. They reported blood loss of 56.7 ± 76.0ml in the NOSES group and 79.0 ± 92.9 ml in the conventional laparoscopic-assisted resection. The robotic technique enhances the accuracy, flexibility, and precision of the NOSES procedure. Zhou et al. (15) reported that 21 (10.1%) patients developed anastomotic leakage (AL) following laparoscopic resection with transrectal NOSE. The narrow pelvic cavity increased the complexity of the transrectal NOSE, such as intracorporeal resection, specimen extraction, rectal stump closure, and intracorporeal anastomosis. Furthermore, Park et al. (16) reported that rectal transection and anastomosis were more difficult with a laparoscopic approach than with open surgery, as the currently available laparoscopic devices are not specifically designed for all types of rectal cancers, especially for narrow pelvic cavities. Besides, the traditional double-stapled technique creates at least two intersecting staple lines, resulting in bilateral stapled corners (“dog ears”), which could increase the incidence of postoperative anastomotic leakage (17). This study reported only two cases of anastomotic leakage, one in the NOSES group and one in the CRR group. The circular stapling single anastomosis realized end-to-end anastomosis without “dog ear” formation and fully restored the structural integrity of the colon. Q. Feng et al. reported that the flexible robotic arms operate accurately and can easily perform suture operations even on the pelvic floor, which improves the quality of the surge (18). The 360° rotating robotic needle holder was convenient and swift for running suture and purse-string suture. According to Sciuto et al. (19), level of vascular ligation could affect the blood supply to the anastomosis, and subsequently anastomotic healing. Preservation of the left colic artery (LCA) increases blood supply to the anastomosis following anterior resection. In this study, the surgeons preserved the LCA and freed the splenic flexion in cases of tension.

This study showed that T4 stage cases accounted for 34.3% in the CRR group and 20.5% in the NOSES group, which is inconsistent with the recommendations of the International consensus on natural orifice specimen extraction surgery (NOSES) for colorectal cancer (10). However, we believe that for experienced teams in colorectal minimally invasive surgery, robotic NOSES for T4 stage cases is safe while strictly observing the principle of tumor-free technique, which we have proved in our previous study (9).

Furthermore, there were no significant differences in the 3-year OS and DFS between the two groups. Two patients in the NOSES group developed local recurrences, including one case of abdominal pelvic cavity recurrence (pT4N0, CDmax: 4.0cm) and another case of local lymph node metastasis (pT3N2, CDmax: 3.5cm). Consistent with this finding, Kim et al. (20) reported one case of multiple regional lymph node metastases and pelvic lymph node recurrence in the NOSES group from a patient who had a 4.5cm tumor. However, it was unclear whether the local recurrence or metastasis was due to the advanced primary tumor or the operation as all included patients had T4 tumors or N+. Taken together, there were no significant differences in the long-term survival outcomes between the robotic NOSES and CRR groups, consistent with a previous study on robotic colorectal surgery (21).

This study had several limitations. First, this was a single-center retrospective study. Therefore, the study could have a selection bias. Second, despite the surgeons being highly experienced in robotic surgeries, they could still make technical errors.

## Conclusion

Robotic natural orifice specimen extraction surgery has several short-term advantages over conventional robotic resection, including less pain, less blood loss, and faster recovery of gastrointestinal function. The unique advantages of robotic systems make NOSES more feasible. Furthermore, robotic NOSES have comparable long-term survival outcomes with CRR.

## Data availability statement

The original contributions presented in the study are included in the article/supplementary material. Further inquiries can be directed to the corresponding author.

## Ethics statement

The studies involving human participants were reviewed and approved by The Ethics Committee of the Second Xiangya Hospital, Central South University. The patients/participants provided their written informed consent to participate in this study.

## Author contributions

LL and HY conceived and designed the article; HY, TL, KL, and SX performed the operation; LL, ZG, SX recorded the information of patients and collected the data; LL and NY analyzed the data; LL wrote the study; HY, KL, JZ, and ZG revised the article. LL should be regarded as the first author. All authors reviewed the manuscript. All authors contributed to the article and approved the submitted version.
